# Composition of Secret Remedies

**Published:** 1878-01

**Authors:** 


### Composition of Secret Remedies.

Professor Richter states that, among nine hundred and thirty-eight secret remedies analyzed by him, he found (1) 22 per cent.

contained substances of violent or poisonous action; and (2) 25 per cent. which, although less active, yet were possessed of medicinal power; while (3) 52 per cent. were of no importance, or quite inoffensive. The first category, especially, comprises violent and poisonous agents for the skin and hair; opiates for children, capable of inducing chronic cerebral disease, or even death; "purifiers of the blood," composed of arsenic or mercury; and a whole legion of violent purgatives, capable of doing, in appropriate cases, an immense amount of mischief. The third category comprises preparations which have nothing in common with the noxious and poisonous effects produced by those of the first and second, but yet agree with them in being sold at from five to a hundred times their proper value, and thus constituting robberies. All these attacks on the public health and morals take place with the full cognizance of the public authorities.—*Med. and Surg. Reporter.*

